# Cracking it - successful mRNA extraction for digital gene expression analysis from decalcified, formalin-fixed and paraffin-embedded bone tissue

**DOI:** 10.1371/journal.pone.0257416

**Published:** 2021-09-16

**Authors:** Alireza Saraji, Anne Offermann, Janine Stegmann-Frehse, Katharina Hempel, Duan Kang, Rosemarie Krupar, Christian Watermann, Danny Jonigk, Mark Philipp Kühnel, Jutta Kirfel, Sven Perner, Verena Sailer

**Affiliations:** 1 Pathology of the University Hospital Schleswig-Holstein, Campus Luebeck, Luebeck, Germany; 2 Research Center Borstel, Leibniz Lung Center, Borstel, Germany; 3 Institute of Pathology, Hannover Medical School, Hannover, Germany; 4 Biomedical Research in Endstage and Obstructive Lung Disease (BREATH), German Center for Lung Research, Hannover, Germany; Universita degli Studi di Milano-Bicocca, ITALY

## Abstract

With the advance of precision medicine, the availability of tumor tissue for molecular analysis has become a limiting factor. This is particularly the case for bone metastases which are frequently occurring in cancer types such as prostate cancer. Due to the necessary decalcification process it was long thought that transcriptome analysis will not be feasible from decalcified formalin-fixed, paraffin-embedded (DFFPE) in a large manner. Here we demonstrate that mRNA extraction from DFFPE is feasible, quick, robust and reproducible and that decalcification does not hamper subsequent gene expression analysis. This might assist in implementing transcriptome analysis from DFFPE into every day practice.

## Introduction

Prostate cancer (PCa) is the most frequent non-cutaneous cancer among men [1]. Although the prognosis of PCa has continually been improving during the last twenty years [2, 3], a significant number of patients will experience tumor progression with metastatic disease and cancer-related death. For example, in 2019, more than 30,000 deaths were caused by metastatic PCa in the United States [1]. The most common site for metastatic spread of PCa is the skeletal system [4, 5]. Bone metastases cause high morbidity with pain and skeletal-related events such as pathological fractures [6]. In the context of precision medicine (PM) recent advances were made in understanding the molecular biology and pathology of cancer by implementing high throughput gene sequencing methods and integrative molecular analyses [7]. The molecular landscape of metastatic prostate cancer differs significantly from primary PCa, therefore molecular analysis from metastases rather than primary tumors might provide the most useful information to guide clinical management [8]. However, availability of metastatic tissue for molecular analyses is considered a major limiting factor, particularly in the setting of tissue obtained from bone metastases [9]. Several biobanking protocols for fresh tissue from PCa bone metastases (PCaBM) have been developed. However, the majority of PCaBM are obtained by routine surgery followed by decalcification [10, 11]. In order to prepare a proper paraffin section from bone tissue, the tissue is generally treated either with acids (formic acids) or with an organic chelating agent such as ethylenediaminetetraacetic acid (EDTA) to soften the bone tissue by reacting with calcium in a process called “decalcification” [12]. Decalcification can result in severe degradation of RNA [13]. The quality of archival FFPE tumor tissue is further affected by several factors including pre-fixation time and process, fixation processing, temperature and sample size. In addition, the quality and quantity of FFPE-extracted RNA is influenced by fragmentation, degradation, and cross-linking with proteins [14, 15]. The resulting low quality and quantity in particular of RNA is thought to hamper further analysis [16]. In a comprehensive study, Bohmann *et al*. compared different RNA extraction methods and could show that the fully automated bead-based method provided the overall best yield and reproducibility for high-throughput RNA expression analysis [11]. Most RNA extraction methods from FFPE (mainly using PCR) were performed from non-bone tissue rather than decalcified bone tissue [17]. Traditionally, quantification of RNA yield analyses was measured by RT-PCR. In contrast, performing digital expression profiling by NanoString™ technology enables RNA quality control without any amplification or enzymatic reactions methods [18–20]. We aimed to show that mRNA extraction from DFFPE bone samples is feasible in a quick, robust and reproducible manner. In addition, subsequent successful digital gene expression (DEG) analyses opens new opportunities to carry out molecular analyses from DFFPE bone samples.

## Materials and methods

### Ethic statement

Our study was approved by the Ethics Committee of the University of Luebeck (project code 18–053, date of approval: March 2^nd^ 2018, date of amendment: June 17^th^ 2020).

### DFFPE samples and cohort description

This study included 36 DFFPE blocks from PCaBM (12), plasma cell myeloma (MM) (12) and normal bone tissue (12) (Table 1). The latter two were used as control tissue. All samples have been collected from the archive of the Institute of Pathology, University Hospital Schleswig-Holstein (UKSH) Luebeck, Germany. All samples have been decalcified using an EDTA based method with a fixation time of 48 to 72 hours. Histopathological evaluation and annotation of tumor areas for macrodissection was performed by Verena Sailer and Anne Offermann.

**Table 1:** Data set reference and cohorts (source of data).

Cohort /Operator-derivated data	Plasma Cell myeloma (Number of samples)	Normal Bone (Number of samples)	PCa Bone metastasis (Number of samples)
Data from Qubit^TM^	12	12	12
Data from NanoDrop®	12	12	0
Data from NanoString™	12	12	12

Study cohort included 36 DFFPE randomly selected blocks from prostate cancer bone metastases (PCaBM) (n = 12), plasma cell myeloma (MM) (n = 12) and normal bone tissue (n = 12).

### RNA extraction from decalcified FFPE blocks

DFFPE blocks from PCaBM, MM and normal bone tissue were sectioned into 8 μm-thick cuts. Two tissue sections were placed on each slide. The sections were compared with the annotated HE and only the marked tumor tissue was scraped off with a scalpel. RNA was isolated using the automatic bead-based Maxwell^®^ RSC RNA FFPE Kit (Cat. No: AS1440, Promega). According to the manufacturer’s manual, the scraped tissue was transferred into a RNase-free tube with 300μl mineral oil and vortexed for 10 seconds. Then the sample was heated at 80°C and incubated at room temperature for a while. Furthermore, 250 μl of master mix including lysis buffer, proteinase K and blue dye was added to the sample and followed by 20 second centrifuging. The sample was later heated at 56°C and 80°C. After an incubation time a DNase cocktail was added and the sample was transferred to the Maxwell^®^ FFPE cartridge yielding purified RNA. The isolated RNA was eluted in 50μl of nuclease-free water and then measured with NanoDrop^®^ or Qubit^TM^. For long-time storage, the RNA samples were divided into 7 μl aliquots and stored at -80°C.

### mRNA quantity and purity assay

The quality and quantity of the extracted mRNA were analyzed by two independent methods to enable inter-operator variability comparison, namely by Qubit^TM^ 2.0 fluorometer (Thermo Fisher Scientific Inc.) and NanoDrop^® (^Thermo Fisher Scientific Inc.). NanoDrop^®^ performs nucleic acid purity and quantification assay by measuring the ratio of absorbance at 260/280 nm. Qubit^TM^ 2.0 utilizes fluorescent dyes to measure the concentration of nucleic acids by determining the emission of relative fluorescence emission (RFU) value for each sample automatically. Since no difference in inter-operator variability was observed between these two methods we continued using the Qubit^TM^ 2.0 fluorometer.

### Digital quality control (QC) NanoString^TM^ analysis for mRNA expression

Digital quality control (QC) analysis for mRNA was performed using the NanoString^TM^ PanCancer Progression Panel. The samples were loaded (10–35 ng RNA in a total of 30 μl loading mixture) on a cartridge and proceeded by utilizing a fully automated Prep Station following manufacturer’s recommendations (NanoString^TM^ Inc.). The proceeded cartridge was then sent for digital analysis with the nCounter^®^ Sprint Profiler system (performed at the Institute of pathology, Hannover Medical School, Hannover, Germany). Data were exported as reporter code count (RCC) files and then imported to NanoString nSolver™ analysis software v4.0 for further analysis. Automatic quality control of mRNA was performed according to the software’s instructions.

### Statistical analyses

All the sample sizes are mentioned in each figure. For comparison of two samples *student’s two-tailed t-test* was used. For comparison of more than two samples *1- way-ANOVA* with Tukey post hoc test was used. A *p<0*.*05* was considered as statistically significant. Data are shown as means ± SD. For statistical analysis and data presentation the following software systems were applied; Qubit^TM^ 2.0 IQ Analyzer, NanoString nSolver™ analysis software v4.0 and Prism 6 (GraphPad Software Inc., San Diego, USA).

## Results

### Quantification and purity assay from DFFPE of MM and normal bone tissue using NanoDrop^®^ operator

**Fig 1:**
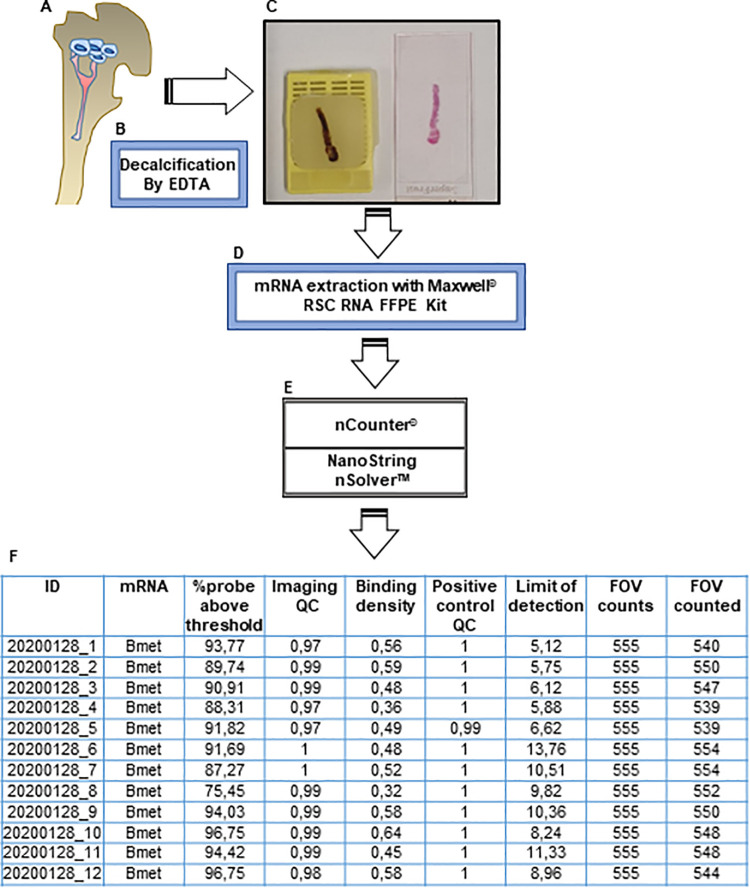
Schematic overview of the workflow. (A) Collecting tissue from PCaBmet patients (B) Decalcification procedure using EDTA (C) Representative picture of DFFPE from PCaBmet tissue (D) mRNA extraction using bead-based Maxwell^®^ RSC RNA FFPE Kit (E) Digital QC by nCounter^®^ reader and nSolver™ analysis software provided by NanoString^TM^ (F) Data output from digital QC nSolver™ analysis software.

**Fig 2:**
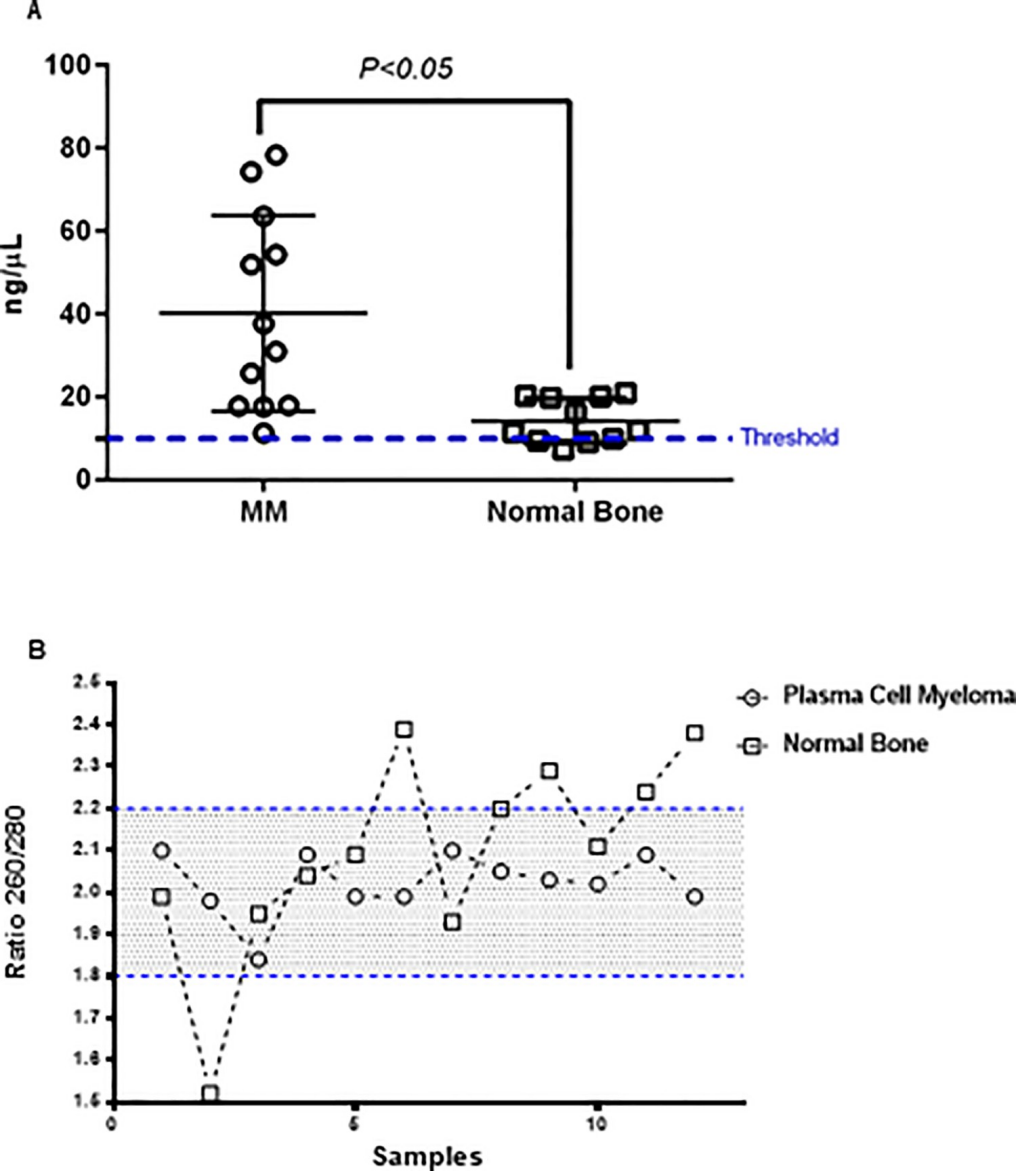
Quantification and purity assay of mRNA yield from DFFPE of normal bone tissue and MM using NanoDrop^®^. (A) Scatter-dots plot indicating quantification of mRNA concentration yield from DFFPE of normal bone tissue and MM using NanoDrop^®^. Dashed line indicates the threshold value for minimum amount of required mRNA for NanoString^TM^ analyses. *p<0*.*05* for comparison, n = 12 for in each group. Data were analyzed by *Student’s t* test and shown as means ± SD. (B) Scatter-line plot representing mRNA qualification by using NanoDrop^®^. Dashed line indicates the threshold value for minimum and maximum ratio (260/280) of different absorption spectra as a measure of mRNA purity extracted from DFFPE of MM and normal bone tissue.

First, we measured the extracted RNA by using NanoDrop^®^ to compare RNA concentration from DFFPE in normal bone tissue and MM. We obtained a satisfactory amount of RNA concentration from both groups above the minimum required RNA concentration for NanoString^TM^ (~10 ng/μL) analyses (Fig 1A–1F). However, the yield of RNA in MM was significantly higher than normal bone tissue (Fig 2A). This was to be expected as the cellularity of MM bone samples is in general higher than in normal bone. The purity of the extracted mRNA was measured as ration ratio of 260/280 of the different absorption spectra and we observed that almost 100% of MM and more than 60% of the RNA from normal bone were indicated as pure RNA (Fig 2B).

### Operator-dependent variability of mRNA yield from DFFPE of MM and normal bone tissue using NanoDrop^®^ and Qubit^TM^

To check the variability between two operators, we performed another mRNA quantification using a Qubit^TM^ 2.0 fluorometer and compared these results with the NanoDrop^®^ results for mRNA extracted from the same samples (MM and normal bone tissue).

**Fig 3:**
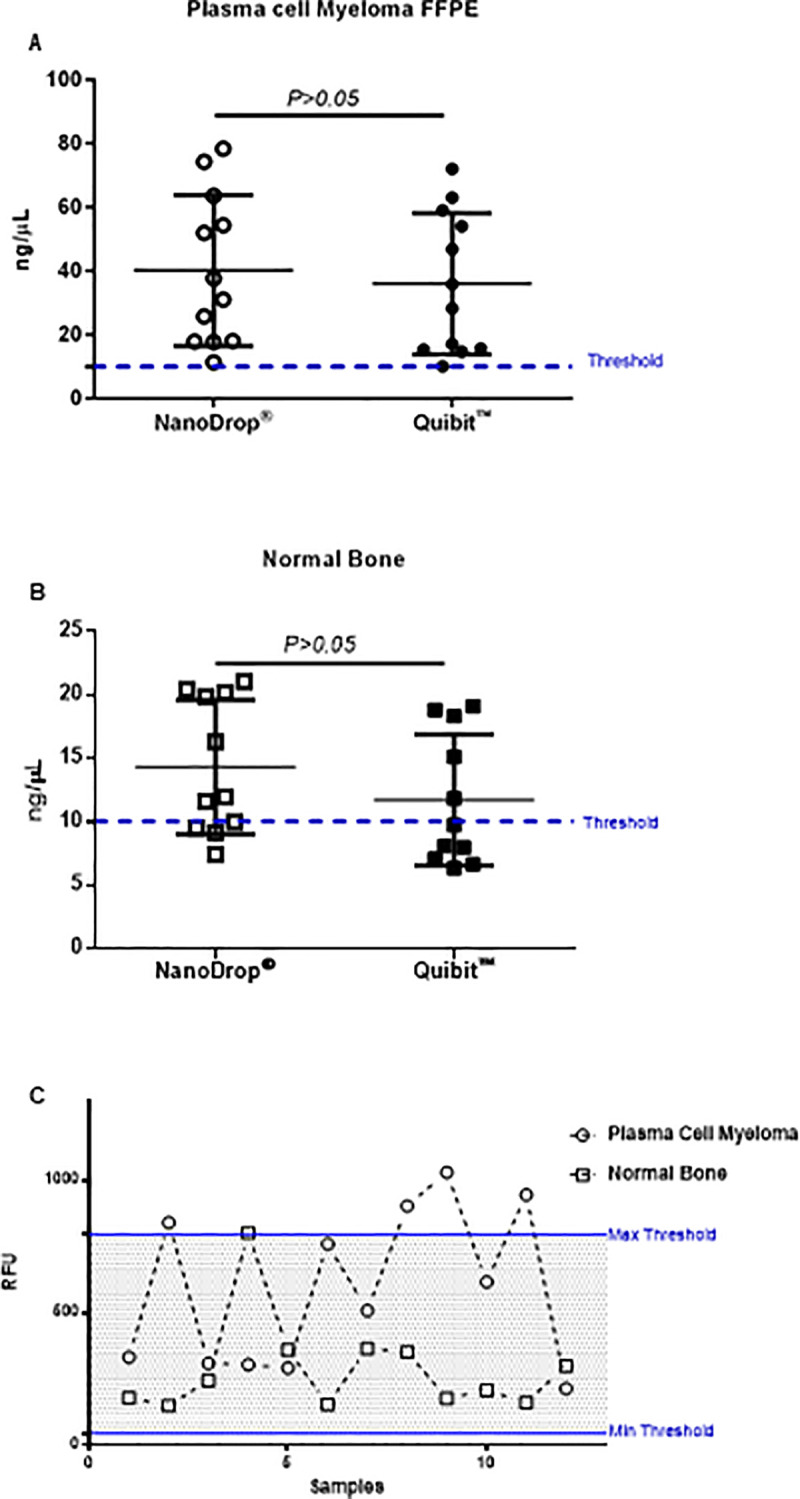
Comparison of mRNA yield from DFFPE of MM and normal bone tissue using NanoDrop^®^ vs. Qubit^TM^. (A) Scatter-dots plot indicating quantification of mRNA concentration yield from DFFPE of MM by using NanoDrop^®^ and Qubit^TM^ 2 Fluorometer. n = 12 for in each group. Data were analyzed by *Student’s t* test *(p>0*.*05)* and shown as means ± SD. (B) Scatter-dots plot indicating quantification of mRNA concentration yield from DFFPE of normal bone tissue by using NanoDrop^®^ and Qubit^TM^ 2 Fluorometer. n = 12 for in each group. Data were analyzed by *Student’s t* test *(p>0*.*05)* and shown as means ± SD. Threshold lines in plot A and B (dashed lines) indicated the minimum required mRNA concentration (~10 ng/μL) for gene expression assay by NanoString^TM^ (C) Scatter-Line plot using Qubit^TM^ 2 Fluorometer representing relative fluorescence emission (RFU) of mRNA extracted from DFFPE of normal bone tissue and MM. lines indicates the threshold for minimum and maximum RFU value.

We found that all samples reached mRNA concentrations as required by NanoString™ (minimum of ~10 ng/μL). In addition, there was no significant difference in RNA concentrations (*p>0*.*05*) by using Qubit^TM^ 2.0 fluorometer or NanoDrop^®^ (Fig 3A and 3B). By monitoring the relative fluorescence emission (RFU) value for each sample using Qubit^TM^ we observed that almost 80% of the extracted mRNA from MM and more than 90% of the mRNA from normal bone tissue were in the standard range of RFU (Fig 3C).

### Quantification of mRNA yield from DFFPE of PCaBM tissue using Qubit^TM^

**Fig 4:**
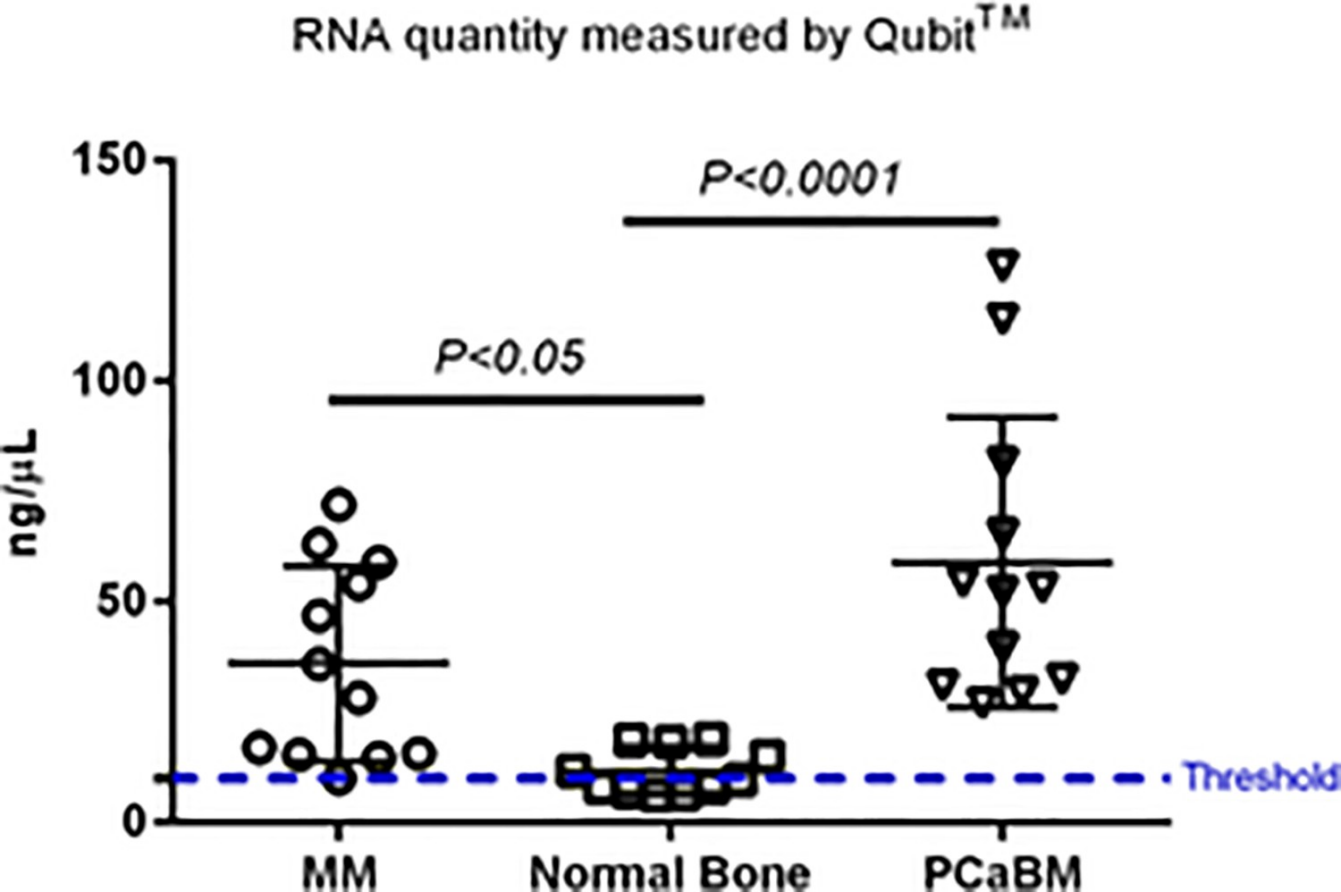
Quantification of mRNA yield from DFFPE of MM, normal bone tissue and PCaBM tissue using Qubit^TM^. Scatter-dot plot indicating quantification of mRNA concentration yield from DFFPE of MM, normal bone tissue and PCaBM tissue using Qubit^TM^ 2 Fluorometer. *p<0*.*05* and *p<0*.*0001* for comparison, n = 12. Data were analyzed by *1-way ANOVA* test and shown as means ± SD. Threshold line indicated the minimum required mRNA concentration (~10 ng/μL) for gene expression assay by NanoString^TM^.

Based on the results from Fig 2, we performed another mRNA measurement for the mRNA extracted from PCaBM tissue using Qubit^TM^ and then compared those to the previous mRNA measurements extracted from DFFPE of MM and normal bone tissue. The quantity of mRNA extracted from PCaBM DFFPE was notably high and above the threshold amount (Fig 4).

### NanoString™ digital quality control

**Fig 5:**
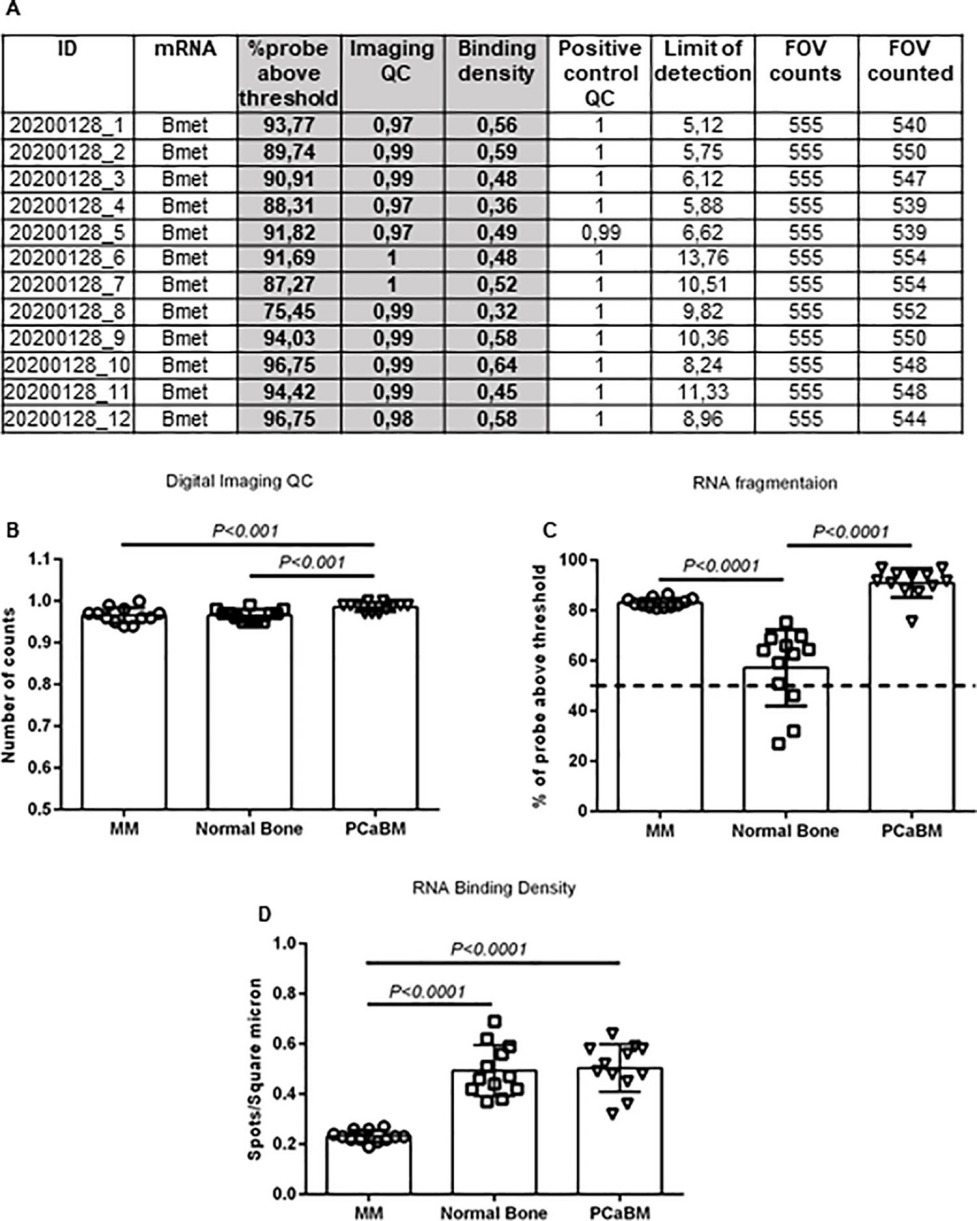
Digital QC analysis of mRNA yield from DFFPE of MM, normal bone and PCaBM tissue using NanoString^TM^ technology. (A) Representative data output of QC nSolver™ analyses for mRNA analyses from DFFPE of PCaBM. (B) Scatter-Bar graph representing digital imaging QC nSolver™ analysis (% of fields of view) of mRNA extracted from DFFPE of MM, normal bone and PCaBM tissue provided by NanoString^TM^. *p<0*.*001* for comparison, n = 12 for in each group. Data were analyzed by *1-way ANOVA* test and shown as means ± SD. (C) Scatter-Bar graph representing mRNA fragmentation by QC nSolver™ analysis. % of probes with more than 300 nucleotides (nt) from DFFPE of MM, normal bone and PCaBM tissue provided by NanoString^TM^. Dashed line indicates the threshold value (50%) for minimum percentage of probes (greater than 300 nt). *p<0*.*0001* for comparison n = 12 for in each group. Data were analyzed by *1-way ANOVA* test and shown as means ± SD. (D) Scatter-Bar plot indicating mRNA binding density using QC nSolver™ analysis from DFFPE of MM, normal bone and PCaBM tissue provided by NanoString^TM^. Dashed line indicates the threshold value for minimum amount of RNA binding density (0.1–1.8 spots per square micron) according to NanoString^TM^. *p<0*.*0001* for comparison n = 12 for in each group. Data were analyzed by *1-way ANOVA* test and shown as means ± SD.

NanoString™ performs an automated quality control of the input mRNA (Fig 5A). Digital imaging QC reports the percentage of field of views (FOVs) which have been successfully scanned by nCounter^®^. Based on the nSolver analysis software user manual at least 75% (0.75) of FOVs must be captured (https://www.nanostring.com/products/analysis-software/nsolver). In this study imaging QC results indicated that all samples passed with a score of minimum 0.9 (90%) thus confirming a robust RNA quantity (Fig 5B). NanoString™ provides another QC for RNA quality evaluation by measuring RNA fragmentation percentage. Based on NanoString^TM^ QC at least 50% of the sample must be more than 300 nucleotide (nt) in length as the optimal quality performance (https://www.nanostring.com/products/analysis-software/nsolver). Our results showed almost all RNA samples are higher than 50%, thus representing a good quality of mRNA (Fig 5C). Binding density is another important parameter of QC and indicates the concentration of barcodes spots (mRNA) per square micron [21]. Our results on mRNA binding density showed that all samples are in the ideal range between 0.1–2.25 spots per square micron (Fig 5D). However, the binding densities of samples from normal bone and PCaBM were significantly higher than from MM *(p<0*.*0001)*.

## Discussion

In this study we were able to demonstrate that mRNA extraction and subsequent digital gene expression analysis using the NanoString™ method from decalcified formalin-fixed and paraffin-embedded bone samples is feasible and produces robust results. Despite well-known RNA degradation in FFPE [22, 23], we have shown that mRNA from DFFPE using an automated bead-based extraction method was sufficient both in quantity and quality to pass the digital QC as provided by NanoString™ technology. Thus, decalcification by EDTA does not hamper subsequent gene expression analysis. In particular, PCaBM showed less mRNA fragmentation and more density equities. Importantly, we did not alter regular mRNA extraction protocols thus confirming that mRNA extraction form DFFPE can be employed in daily routine. We initially started with utilizing tissue from PCaBM for gene expression analysis to study the complex landscape of metastatic prostate cancer. Even though we employed archival DFFPE that was several years old we successfully performed NanoString™ analyses. This opens up new opportunities for gene expression analysis in the daily management of patients e.g., for patients with hematological diseases who usually undergo routine bone biopsy at the time of first diagnosis and often during therapy as well. The turn-around time from macrodissection to data analysis is around 6–10 working days. This short timespan might further assist implementing gene expression analysis from DFFPE in clinical management. Furthermore, the vast archives of pathology laboratories worldwide [24] provide a valuable, as yet largely untapped resource for studying bone metastases, benign bone diseases and the bone microenvironment. Our work was limited by the sample’s range and number. We are confident that this initial study warrants performing large scale transcriptomic analyses from bone samples.

## Conclusions

In conclusion we have shown that DFFPE can be utilized for gene expression analysis thus assisting to integrate transcriptome data into everyday patient care to improve the prognosis and prediction.

## List of abbreviation

PCa: Prostate cancer
PM: Precision medicine
PCaBM: Prostate cancer bone metastases
DFFPE: Decalcified formalin-fixed, and paraffin-embedded
EDTA: Ethylenediaminetetraacetic acid
FFPE: Formalin-fixed paraffin-embedded
DEG: Differentially expressed genes
DSP: Digital spatial profiling
MM: Plasma cell myeloma
RFU: Relative fluorescence unit
IQ: Integrity and quality
QC: Quality control
RCC: Report code count
FOV: Field of view
IHC: Immunohistochemistry
NGS: Next generation sequencing
H&E: Haematoxylin and Eosin
